# Lycopene-Loaded Bilosomes Ameliorate High-Fat Diet-Induced Chronic Nephritis in Mice through the TLR4/MyD88 Inflammatory Pathway

**DOI:** 10.3390/foods11193042

**Published:** 2022-09-30

**Authors:** Chang Liu, Yu Liu, Ciwan Wang, Yahui Guo, Yuliang Cheng, He Qian, Yong Zhao

**Affiliations:** 1 State Key Laboratory of Food Science and Technology, Jiangnan University, No.1800 Lihu Avenue, Wuxi 214122, China; 2 Wuxi 9th People’s Hospital Affiliated to Soochow University, Wuxi 214122, China; 3 Thoracic and Cardiac Surgery, Affiliated Hospital of Jiangnan University, No.1000, He Feng Road, Wuxi 214122, China

**Keywords:** lycopene, chronic nephritis, TLR4/MyD88, metabolomics

## Abstract

Chronic kidney disease caused by a high-fat diet (HFD)-induced metabolic syndrome has received widespread attention. Lycopene has a wide range of biological activities and can improve a variety of chronic diseases through anti-inflammatory effects. In this study, HFD-fed mice were used as a metabolic syndrome model to evaluate the protective effect of lycopene in a sustained-release vehicle (bilosomes) in the small intestine against renal injury and to determine whether the TLR4/MyD88 pathway and related metabolic pathways are involved in this process. The results showed that lycopene bilosomes alleviated HFD-induced kidney damage, as evidenced by lower serum urea nitrogen, creatinine, and uric acid levels. Histopathology studies showed that lycopene bilosomes attenuated HFD-induced tubular cell and glomerular injury. In addition, Elisa, RT-PCR, and Western blotting results showed that lycopene bilosomes also reduced the expression of inflammatory factors such as TLR4, MyD88, NF-kB, TNF-a, and IL-6 in mouse kidneys. The mechanism was to attenuate renal inflammatory response by inhibiting the TLR4/MyD88 inflammatory pathway. These findings suggested that lycopene can alleviate nephritis and metabolic disorders caused by HFD, inhibiting the TLR4/MyD88 inflammatory pathway and its downstream pro-inflammatory cytokines and further regulating the vitamin K metabolism, beta-alanine metabolism, and glutathione metabolism pathways to relieve chronic nephritis.

## 1. Introduction

A high-fat diet (HFD) leads to lipid accumulation in the liver, contributing to the progression of chronic metabolic diseases, such as chronic nephritis and atherosclerosis [1,2]. Mice can be used as models for atherosclerosis and may also help elucidate the mechanism by which HFD affects nephritis [3]. A study found that HFD significantly increased the production of reactive oxygen species, which released superoxide anion and caused oxidative stress and inflammation in the kidney by stimulating podocyte damage [4]. Notably, nephritis is a common condition that leads to renal failure and related diseases. HFD affects peripheral tissues and causes systemic inflammation through direct or indirect activation of the Toll-like receptor 4 (TLR4)/myeloid differentiation primary gene 88 (MyD88) pathway [5]. Most TLRs have been reported to use an adaptor molecule, the protein product of MyD88, the absence of which alters TLR4 downstream signaling [6]. When the TLR pathway is activated, nuclear factor kappa-B (NF-kB) is subsequently activated and translocated to the nucleus to induce various inflammatory cytokines, such as interleukin-1β (IL-1β), interleukin-6 (IL-6), and tumor necrosis factor-a (TNF-a) [7]. Although these pro-inflammatory cytokines continue to mediate inflammation and exacerbate kidney disease, hypoglycemic and lipid-lowering drugs may help inhibit the progression of nephritis [8]. However, a growing body of research suggests that clinical drugs may have unwanted side effects [9]. Therefore, it is significant to alleviate the inflammatory response caused by HFDs.

Lycopene, a bioactive component mainly found in foods such as tomatoes, is characterized by various biological activities such as scavenging reactive oxygen radicals and anti-inflammatory and neuroprotective functions [10]. It is the most potent oxygen-free radical quencher of all the natural carotenoids and has been linked to protection against cardiovascular diseases, inflammation, and many cancer types [11]. A study finds that lycopene can also relieve kidney dysfunction caused by toxic substances [12]. However, few studies have been able to determine whether and how lycopene reduces renal cell damage caused by HFDs. Hydrophobicity of carotenoids determines their metabolic fate in the gastrointestinal tract—poor stability and low bioavailability [13]. At the same time, carotenoids are sensitive to environmental conditions such as temperature, oxygen, and light, posing problems in their widespread applications. Thus, developing effective delivery systems is crucial in improving their bioavailability and functions [14].

Nanoparticle delivery systems, including nanoemulsions and nanoliposomes, are excellent vehicles to increase carotenoid stability and bioavailability in the small intestine [15]. Vesicle carriers such as bilosomes, formed by incorporating bile salts into the lipid bilayer, serve as stable replacements for conventional vesicles in the digestive environment of the small intestine [16]. They provide higher bioavailability of active agents as they better protect them from gastrointestinal bile salts and enzymes [17]. Moreover, the bilosomes added with sodium deoxycholate showed longer residence time in the gastrointestinal tract with higher intestinal permeability, improving the therapeutic effect on non-alcoholic fatty liver disease in mice [18]. Therefore, there is a huge opportunity in using bilosomes as lycopene transport vehicles, especially for sustained release and absorption in the small intestine.

However, the sustained-release effect and bioavailability of lycopene-loaded bilosomes in the small intestine remains unknown. Therefore, the purpose of this study was to use the bilosomes to deliver lycopene in the small intestine in a sustained-release manner, interfering with HFD-induced chronic nephritis in mice. Further, we also studied the effect of lycopene on serum and renal inflammatory indicators in mice and, finally, explored the effect of lycopene on the interventional effect of TLR4/MyD88 and metabolic pathways in mouse kidneys on chronic nephritis.

## 2. Materials and Methods

### 2.1. Materials

Lycopene, lecithin, sodium cholesterol deoxycholate, and Tween80 were purchased from Sinopharm Chemical Reagent Co., Ltd. (Shanghai, China). Other reagents were of analytical grade. Blood glucose test strips were purchased from Roche Biotechnology Co., Ltd. (Shanghai, China).

### 2.2. Preparation of Small Intestinal Sustained-Release System

References for the preparation process of bilosomes are found in [17,19]. The sample preparation process of bilosomes is by dissolution of raw materials, rotary evaporation, and sonication. lycopene (30 mg), lecithin (300 mg), Tween 80 (100 mg), and sodium deoxycholate (75 mg) were thoroughly dissolved in a 50 mL round-bottom flask containing a small amount of chloroform. The mixture was dried by rotary evaporation (removal of organic solvents) at 45 °C. The residue was further dried in vacuo (at 45 °C) to ensure complete removal of organic solvent, then 10 mL of phosphate-buffered saline (PBS) solution (pH 7.4) was added and hydrated at 45 °C with vortexing for 60 min. The bilosome suspension was sonicated (240 W, kHz, 10 min) in an ice bath. Finally, bilosomes were stored in the refrigerator (4 °C, protected from light) until use.

### 2.3. Animal Experiments

**Figure 1:**
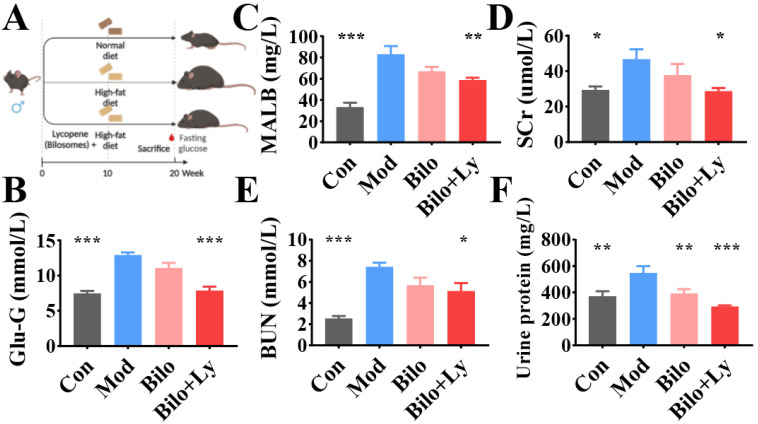
The effect of lycopene on the biochemical analysis of mouse serum. Animal experiment flow chart (**A**). Fasting blood glucose levels in mice (**B**). MALB content in serum (**C**). SCr content in serum (**D**). BUN content in serum (**E**). Urine protein content in serum (**F**). Signs of significance of difference between Mod group and other groups: * *p* < 0.05, ** *p* < 0.01, *** *p* < 0.001.

As shown in Figure 1A, 24 ApoE-/-C57BL/6J mice (male, 6 weeks old) (Shanghai, Nanjing) were provided by GemPharmatch Co., Ltd. (Nanjing, China). Animal ethics was approved by the Institutional Animal Care and Use Committee of Jiangnan University JN. No. 20211030c1400210 (413). Mice were housed in an environment with stable temperature (25 ± 2 °C) and humidity (60 ± 10%). After 7 days of adaptive feeding of the basal diet, the mice in the control group were fed a high-fat and high-cholesterol diet, excluding the Con group. At week 10, mice were orally gavaged with samples (Bilo, Bilo+Ly) at a dose of 50 mg/kg. At the same time, the Con group and the Mod group mice were gavaged with normal saline.

### 2.4. Determination of Fasting Blood Glucose and Serum Biochemical Indexes in Mice

The mice were fasted for 12 h on the last day of the experiment, but they were allowed to drink water. After the mice were anesthetized, blood was collected through the tail vein, and the fasting blood glucose (FBG) of the mice was measured with a blood glucose meter (Roche, Switzerland, Accu-Chek). Eyeballs were picked with ophthalmic forceps to collect blood, and the plasma was left to stand at room temperature for half an hour. The plasma was centrifuged at 4 °C and 4000× *g* for 15 min to obtain serum, and the serum was stored at −20 °C for subsequent index detection. Serum indexes such as blood urea nitrogen (BUN) and serum creatinine (SCr) were detected and analyzed by the automatic blood analyzer of Mindray Biomedical Company (Shenzhen, China).

### 2.5. Histopathological Determination of Kidney in Mice

After the blood was collected from the mice, part of the kidney tissue was quickly cut and fixed with 4% paraformaldehyde for 24 h and then taken out. Tissues were sequentially dehydrated with graded alcohol concentrations and then embedded in paraffin. The tissue wax blocks were cooled at −20 °C and cut into 4 μm slices with a microtome. The tissue sections were unfolded in warm water at 40 °C and mounted on glass slides. The slides were taken out after baking at 60 °C and stored at room temperature. Finally, the sections were dewaxed, immersed in hematoxylin and eosin staining solution (H & E) for staining, dehydrated, and mounted with gum. Pathological sections were observed under a light microscope, and images were analyzed.

### 2.6. Determination of Inflammatory Factors in Mice Kidney

After the mice’s blood was collected, 0.2 g of each kidney tissue was quickly cut out, rinsed with pre-cooled normal saline, and then dried with filter paper. Finally, kidney tissue was homogenized at a concentration of 10%. The prepared kidney tissue homogenate was then centrifuged at 3000× *g* at 4 °C for 10 min, and the protein concentration, TNF-α, IL-6, and IL-1B contents in the supernatant were determined according to the instructions. ELISA kits were purchased from Nanjing Senbeijia Biotechnology Co., LTD. (Nanjing, Jiangsu, China).

### 2.7. RNA Extraction and Quantitative Real-Time PCR (qRT-PCR)

Total RNA in kidney tissue was isolated by a kit from Sangon Biotech Co., Ltd. (Shanghai, China) according to the manufacturer’s instructions. Samples with an absorbance ratio at 260/280 nm between 1.8 and 2.0 were judged to be of acceptable quality and integrity. Genomic DNA was removed from RNA using a cDNA reverse transcription kit (Vazyme Biotech Co., Ltd., Nanjing, China), and RNA was reverse transcribed into cDNA. The processes were performed with reference to the SYBR qPCR Master Mix (Vazyme Biotech Co., Ltd., Nanjing, China) kit and the XLX-096D real-time fluorescence quantitative PCR instrument operating instructions (Xin Lanxin Biotechnology Co., Ltd., Jiangsu, China). β-action was used as a reference gene for normalization. Data are presented as relative mRNA levels according to the 2-ΔΔCt method. Refer to Supplementary Table S1.

### 2.8. Western Blotting Analysis

Radioimmunoprecipitation assay buffer containing phosphatase inhibitor and protease inhibitor from Sangon Biotech Co., Ltd. (Shanghai, China) was added to kidney tissue to prepare tissue homogenate. Kidney tissue protein was extracted with a bicinchoninic acid protein extraction kit, and protein concentration was determined according to the manufacturer’s instructions (Beyotime Institute of Biotechnology, Jiangsu, China). Samples were separated by 10% SDS-PAGE and transferred to polyvinylidene fluoride membranes. Membranes were blocked with 5% nonfat dry milk in TBST buffer at 37 °C for 2 h, then polyvinylidene fluoride membranes were incubated with primary antibody overnight at 4 °C. Antibodies were purchased from Proteintech (Wuhan, Hubei, China) and GenScript Corporation (Jiangsu, China). Subsequently, the membrane was incubated with the secondary antibody for 2 h at 37 °C and then washed 5 times with TBST. Subsequently, fluorescence intensity was measured by incubation with an enhanced chemiluminescence reagent (Beyotime Institute of Biotechnology, Nantong, China). Bands were quantified using ImageJ software v1.5 (USA).

### 2.9. Metabolite Extraction and GC–MS Analysis

For sample preparation and gas mass spectrometry analysis, refer to reference [20]. A total amount of 200 mg of mouse kidney tissue was mixed with 1 mL of extraction solution (acetonitrile/isopropanol [1:1, *v*/*v*]). The mixture was vortexed for 10 s and centrifuged at 13,000× *g* for 20 min at 4 °C. Subsequently, 450 μL of the supernatant was transferred to a new 2 mL Eppendorf tube. A total of 10 μL of supernatant was withdrawn from each sample and analyzed as a QC sample. The remaining supernatant was dried in a concentrator, and 80 μL of 20 mg/mL methoxyamine hydrochloride (dissolved in pyridine) was subsequently added to the dried metabolite, mixed, and sealed in an oven at 80 °C for 1 h. After cooling to room temperature, 100 μL MSTFA (containing 1% trimethylchlorosilane, *v/v*) was added to each sample. Samples were then scheduled for GC–MS analysis after a further 1.5 h incubation at 70 °C. A 1 mg/mL fatty acid/methyl ester mixture (FAME; C8–C16: 1 mg/mL; C18–C24: 0.5 mg/mL in chloroform) was used as an internal standard.

### 2.10. Data Analysis

All experiments were repeated at least 3 times, and data were presented as mean ± standard deviation (SD). Data were statistically different by one-way ANOVA in GraphPad Prism 7.0 (GraphPad Prism Software Inc., San Diego, CA, USA) (*p* < 0.05 was considered statistically significant).

## 3. Results

### 3.1. Lycopene-Loaded Bilosomes Improve Serum Renal Indices in Mice

To evaluate the effect of lycopene-loaded bilosomes on blood glucose, the fasting blood glucose (FBG) of mice was measured. Compared with the Control group (Con), the blood glucose level of the mice in the Mod group was significantly increased (*p* < 0.001). Compared with the Mod group, the blood glucose values of mice decreased significantly with lycopene-loaded bilosomes, whereas the bilosomes had no effect (Figure 1B). To evaluate the renal status of each group, the contents of MALB, SCr, BUN, and urine protein were detected. As shown in Figure 1C–F, MALB, SCr, and BUN contents in the Bilo group did not change significantly (*p* < 0.05) in Mod, while BUN, MALB, and SCr contents in Con increased significantly (*p* < 0.001). This indicated that the nephropathy model was successfully constructed. Compared with the Mod group, BUN and SCr contents in Bilo+Ly were significantly decreased (*p* < 0.05). Notably, MALB and urine protein of Bilo+Ly significantly reduced to 33.1 mg/L and 293.01 mg/L, respectively, compared to Mod (83.1 mg/L) (546.8 mg/L) (*p* < 0.01).

### 3.2. Lycopene-Loaded Bilosomes Ameliorate Kidney Pathology in Mice

**Figure 2:**
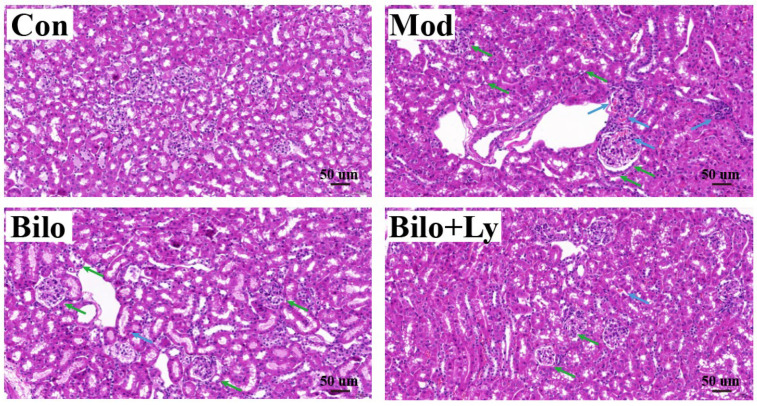
The effect of lycopene on the histopathology of mouse kidney. Histopathology of hematoxylin and eosin staining (200×) of kidney sections from different groups (Con, Mod, Bilo, and Bilo+Ly). Glomerular atrophy is impaired (green arrows), and tubules and blood vessels are dilated (blue arrows).

As shown in Figure 2, hematoxylin and eosin (H & E) staining results showed that no obvious pathological damage was observed in the kidney tissues in the Con group. The structures of renal tubules and glomeruli were clear, the distribution of mesangial cells and the extracellular matrix was normal, and there was no proliferation and fibrosis observed. Moreover, the number of glomerular cells in the Mod and Bilo groups increased, there was no clear space between cells, the cell atrophy was severe, the volume was significantly increased, and the basement membrane was expanded or infiltrated by inflammation. In contrast, the group injected with lycopene contributed to the improvement of morphology. Tissue damage of the mice in the Bilo+Ly group was less than that in the Mod group, the structure of the glomerulus was clear, and the lumen of the renal tubules was regular.

### 3.3. Lycopene-Loaded Bilosomes Improve Nephritis in Mice

**Figure 3:**
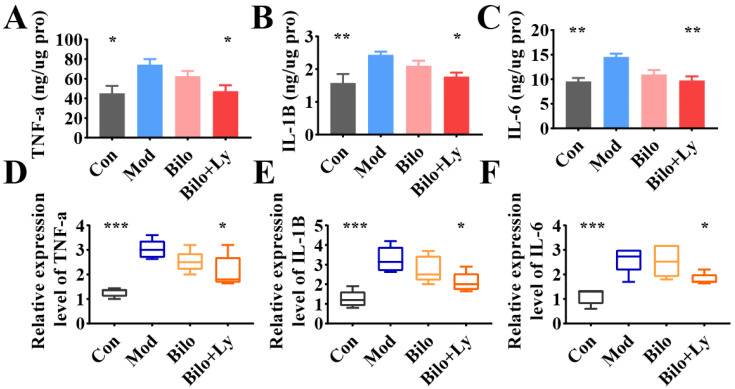
The effect of lycopene on the biochemical analysis of mouse kidney. The content of TNF-a in mouse kidney (**A**). The content of IL-1B in mouse kidney (**B**). The content of IL-6 in mouse kidney (**C**). Expression levels of TNF-a mRNA in mouse kidneys (**D**). The expression level of IL-1B mRNA in mouse kidney (**E**). The expression level of IL-6 mRNA in mouse kidney (**F**). Signs of significance of difference between Mod group and other groups: * *p* < 0.05, ** *p* < 0.01, *** *p* < 0.001.

In addition, the levels of key inflammatory factors in the kidneys were also analyzed. The results of ELISA are shown in Figure 3A–C. The content of TNF-a in the kidney tissue of Mod mice was significantly increased (*p* < 0.05) compared with the Con group. Moreover, IL-1B and IL-6 were significantly increased (*p* < 0.01), indicating that HFD increased the content of inflammatory factors in mouse kidneys. Compared with Mod group, after ten weeks of lycopene-loaded bilosomeadministration, the contents of TNF-a and IL-1B in the kidneys decreased in Bilo+Ly group (*p* < 0.05). Compared to Mod’s IL-6 (14.5 ng/ug protein), lycopene-loaded bilosomes significantly reduced to 9.7 ng/ug protein (*p* < 0.01). Meanwhile, the transcription of inflammatory target genes was analyzed by a qRT-PCR assay (Figure 3D,E). The results showed that under the intervention of HFD, the mRNA levels of TNF-a, IL-6, and IL-1B in Mod mice were significantly up-regulated (*p* < 0.001), and IL-1B expression showed a decreasing trend (*p* < 0.05), whereas there was no significant difference in the Bilo group.

### 3.4. Lycopene-Loaded Bilosomes Inhibit the TLR4/MyD88 Inflammatory Pathway in Mice

**Figure 4:**
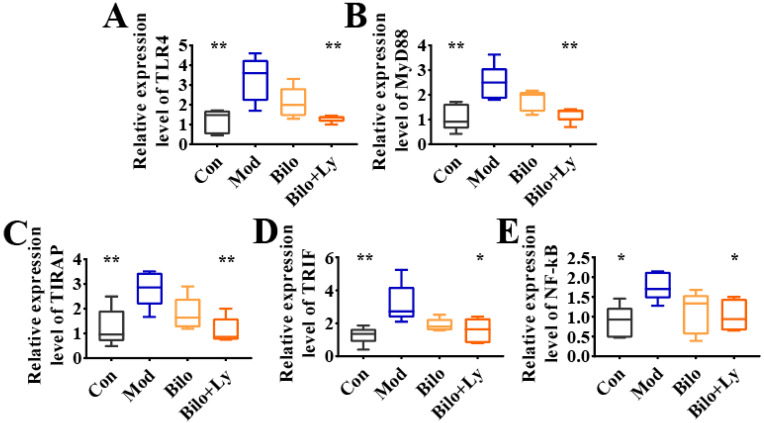
Effect of lycopene on gene expression levels of TLR4/MyD88 pathway. The expression level of TLR4 mRNA in mouse kidney (**A**). The expression level of MyD88 mRNA in mouse kidney (**B**). The expression level of TIRAP mRNA in mouse kidney (**C**). The expression level of TRIF mRNA in mouse kidney (**D**). Expression levels of NF-kB mRNA in mouse kidneys (**E**). TLR4 pathway effect map. Signs of significant differences between the Mod group and other groups: * *p* < 0.05, ** *p* < 0.01.

Accumulated evidence in recent years has shown that the TLR4/MyD88 pathway aggravates the body’s inflammatory response by regulating the expression of a series of inflammatory response genes. To assess the molecular mechanism by which lycopene protects cellular redox state balance, the activation and downstream signaling of the TLR4/MyD88 inflammatory pathways were analyzed. The mRNA levels of TLR4, MyD88, tIR domain-coding adaptor protein (TIRAP), total internal reflection fluorescence (TIRF), and NF-kB in each group were determined by qRT-PCR, and the results are shown in Figure 4A–E. Compared with the Con group, the mRNA levels of inflammatory genes in the Mod group were increased to varying degrees, whereas the transcription of TLR4, MyD88, TIRAP, and TIRF was significantly increased (*p* < 0.01). Further, NF-kB mRNA levels were also significantly increased (*p* < 0.05). In contrast, although the level of inflammatory factors decreased in the Bilo group, it was not significant. However, the intervention of lycopene-loaded bilosomes attenuated this change. The mRNA transcription of TLR4, MyD88, TIRAP, TIRF, and NF-kB were increased to different degrees compared with Mod (*p* < 0.05, *p* < 0.01). These results demonstrate that lycopene reduces the expression of factors in the TLR4/MyD88 inflammatory pathway in the kidneys of HFD mice.

**Figure 5:**
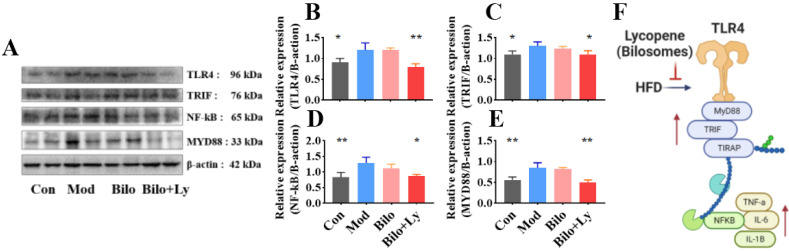
Effect of lycopene on protein expression levels of TLR4/MyD88 pathway. Signaling molecule expression, including TLR4, TRIF, MyD88, and NF-κB (**A**). The expression level of TLR4 in mouse kidney (**B**). The expression level of TRIF in mouse kidney (**C**). Expression levels of NF-kB in mouse kidneys (**D**). The expression level of MyD88 in mouse kidney (**E**). TLR4 pathway effect map (**F**). Signs of significant differences between the Mod group and other groups: * *p* < 0.05, ** *p* < 0.01.

In order to further verify the effect of lycopene on the TLR4 pathway in nephritis mice from the protein level, we investigated the protein levels of TLR4, TRIF, MyD88, and NF-κB. As shown in Figure 5A–E, compared with the Mod group, the protein levels of TLR4, TRIF, MyD88, and NF-κB in the Con group were significantly decreased (*p* < 0.05), and the protein expression in the Bilo+Ly group was also significantly decreased (*p* < 0.05). Among them, the protein levels of TLR4 and MyD88 in the Bilo+Ly group were extremely significant (*p* < 0.01). Compared with Mod, TLR4, TRIF, MyD88, and NF-κB protein concentrations were slightly decreased in the Bilo group, but there was no significant difference between groups. These results are also consistent with those for mRNA.

### 3.5. Lycopene Improves Renal Metabolism in Mice

**Figure 6:**
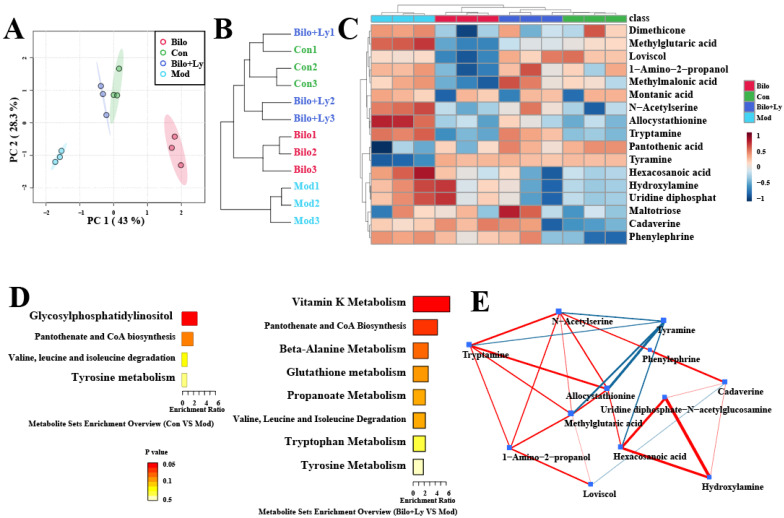
The effect of lycopene on renal metabolism in mice. Metabolite PCA plot (**A**). Metabolite cluster dendrogram (**B**). Differential metabolite heatmap (**C**). Differential metabolic pathway enrichment map (**D**). Differential metabolite network diagram (**E**).

Metabolomics was used to explore the metabolite changes upon lycopene’s effects in mice by qualitative and quantitative small-molecule metabolites in renal tissue. It can be seen from the principal component analysis (PCA) graph and clustering tree (Figure 6A,B) that after the intervention of lycopene, the Mod group and the Con group were significantly different, while the Bilo+Ly group and the Con group were similar. As shown in Figure 6C, the screened differential metabolites were visualized as a heat map (VIP > 1, *p* < 0.05). Compared with the Mod group, lycopene decreased the dimethicone, hexacosanoic acid, and hydroxylamine metabolites, but increased methylglutaric metabolites such as acid, methylmalonic acid, montanic acid, and maltotriose. Clearly, these metabolites are the main markers of lycopene metabolism in mouse kidneys. Therefore, we carried out a functional analysis of the significantly different metabolites to explore the pathways affected, as shown in Figure 6D. In the comparison to Con and Mod, glycosylphosphatidylinositol, pantothenate, and CoA biosynthesis were the more significantly affected pathways. In comparison to Bilo+Ly and Mod, the more obvious pathways were vitamin K, beta-alanine, glutathione, propanoate, pantothenate, and CoA biosynthesis. Finally, the effect of lycopene on the metabolite network was analyzed by a network diagram (Figure 6E); the metabolites with significant positive correlation were uridine diphosphate-N-acetylglucosamine, hexacosanoic acid, and hydroxylamine, while the metabolites with significant negative correlation were tyramine, allocystathionine, and methylglutaric acid.

## 4. Discussion

Lycopene is a diverse biologically active antioxidant and anti-inflammatory agent that has been extensively studied in various disease models, such as arteriosclerosis, diabetes, and cancer [11]. A study shows that the lycopene diet can improve the kidney’s antioxidant capacity by activating Nrf2 expression [21]. Nrf2 is a major transcription factor that regulates cellular oxidative stress responses, controls the primary defense mechanism against reactive oxygen species-induced tissue damage, and induces the expression of cellular inflammatory cytokines, including IL-β, IL-6, and TNF-a [22,23]. At present, kidney disease caused by dietary habits has received extensive attention. In this study, the HFD-stimulated chronic nephritis mice model was used to investigate the protective effect of lycopene-loaded bilosomes on renal inflammation. We found that the levels of FBG, MALB, SCr, BUN, and urine protein in fat-rich diet-fed mice increased. MALB [24], SCr [25], BUN [26], and urine protein [27] are considered hallmarks of nephritis. These findings suggested that HFD has the ability to alter the body’s metabolic homeostasis and serum renal function metabolism, ultimately leading to inflammatory accumulation in renal tissue. Under the intervention of lycopene, the renal function indexes in the serum of the mice were improved, indicating the protective effect of lycopene.

Excessive intake of a fat-rich diet may lead to dyslipidemia and systemic inflammation, ultimately leading to the development of renal inflammation [28]. HFD-induced metabolic abnormalities easily alter the expression of kidney-related genes, especially inflammatory genes, therefore inhibiting renal inflammation. Thus, it is considered a feasible strategy for the treatment of chronic kidney disease [29]. The TLR4/MyD88 pathway has been recognized as a central link in the pathogenic process of systemic inflammation and high-fat exposure-induced kidney injury [30]. In the present study, on the one hand, TLR4/MyD88 activation was found to be significantly up-regulated in HFD-fed mice, resulting in an increase in the expression of pro-inflammatory cytokines and related chemokines, including IL-β, IL-6, and TNF-a. On the other hand, HFD could up-regulate the levels of inflammatory factors in serum, indicating HFD-induced renal inflammation, suggesting that TLR4/MyD88-activated signaling is involved in HFD-induced renal injury. Furthermore, we found that lycopene-loaded bilosomes could protect against HFD-induced kidney injury by inhibiting pro-inflammatory cytokines and chemokines. Notably, lycopene intervention in mice further suppressed the increase in fasting blood glucose and serum renal function indicators, suggesting that lycopene inhibits HFD-stimulated renal metabolic syndrome. Importantly, as discussed above, HFD-induced inflammation may lead to upregulation of TLR4/MyD88 signaling activation, which tends to exacerbate inflammatory responses and oxidative stress. Therefore, the protective effect of lycopene on the inhibition of TLR4/MyD88 activation was determined. At the same time, the qPCR analysis showed that the mRNA levels of TLR4, MyD88, TIRAP, TIRF, and NF-Kb were increased in the mice in the Mod group, indicating that HFD-induced renal injury exhibits an inflammatory role. However, ten weeks of lycopene intervention significantly reduced the mRNA levels of TLR4, MyD88, TIRAP, TIRF, and NF-Kb in the Bilo+Ly group, and there was no significant difference in the Bilo group, which indicated that lycopene inhibited inflammatory cells, improving chronic nephritis.

The study also found that NF-kB, in addition to its role mediated by TLR4, is a transcription factor that plays an important role in inflammation, immunity, cell proliferation, differentiation, and survival [31]. Furthermore, intracellular NF-kB activation has been described as a molecular mechanism by which inflammatory stress leads to renal injury [32]. Meanwhile, physiologically, inflammatory factors such as TNF-a play a major role in the regulation of immune responses, and the association between inflammation is shown to be closely related to abnormal TNF-a expression [33]. Therefore, reducing the expression of NF-kB and TNF-a may be a therapeutic approach to controlling chronic nephritis. The present study found that HFD-induced mice displayed significantly increased NF-kB and TNF-a expression in kidney tissues, suggesting that inflammation-laden kidney cells can induce cellular damage, as shown in pathological sections. Interestingly, lycopene reduced the levels of these inflammatory factors.

Compared with the Mod group, it was found that the mRNA and protein levels of TLR4, MyD88, and TRIF in the kidneys of mice were decreased in Bilo+Ly group by analyzing the downstream of the TLR signaling pathway (Figure 5F). Triggering the TLR pathway leads to branch activation involving the adaptor protein MyD88 [34,35], induces the activation of proteins such as TRIF, and ultimately leads to the translocation of NF-κB to the nucleus [36]. This process may be critical for the inflammatory response of kidney cells [37]. Prevention of inflammation-induced nephropathy in TLR4-/- transgenic mice further supports the idea that these receptors play a central role in the signaling mechanisms of inflammation [38]. The fact that the TLR4/MyD88 pathway is identified as a key factor in the inflammatory renal response supports the stimulation of gene expression by downstream transcription factors, including those encoding TNF-α, IL-6, and other inflammatory cytokines [39]. The results of this study also found that lycopene can alleviate high-fat diet-induced nephritis in mice, mainly by regulating the expression of the TLR4/MyD88 pathway and its downstream inflammatory factors.

Therefore, we speculated that lycopene-mediated renal protection is associated with attenuated inflammation-induced cytotoxicity and contributes to the improvement of blood lipids and blood glucose, as well as the reduction of intrarenal damage. These findings bring new insights into our understanding of the molecular mechanisms linking metabolic disturbances to kidney damage caused by consuming various fat-rich foods.

The metabolome results also indicated that lycopene mainly enhanced vitamin K, beta-alanine, glutathione, and propanoate metabolism in mouse kidneys. Among them, chronic kidney disease is associated with extensive cardiovascular calcification, partly related to vitamin K metabolism [40]. Common metabolic pathways in the kidneys include beta-alanine metabolism. The medicinal fungus, Phellinus linteus, has the effect of protecting kidneys and resisting hyperuricemia, which is mainly related to beta-alanine metabolism [41]. In addition, astragaloside can mediate anti-cisplatin-induced acute kidney injury in rats, and the related renal metabolic pathway also involves glutathione metabolism [42]. It can be seen that the above metabolic pathways are likely to be the key pathways by which lycopene interferes with nephritis.

## 5. Conclusions

In conclusion, this study demonstrates that HFD upregulates oxidative stress and induces systemic inflammation, leading to renal inflammation in mice. After lycopene-loaded bilosome intervention, the expression of inflammatory factors in the serum and kidneys of mice was reduced. Notably, TLR4/MyD88 and NF-kB activation are key targets involved in renal injury. Lycopene alleviated HDF-induced renal inflammation by inhibiting the TLR4/MyD88 inflammatory pathway by blocking inflammation in mice kidneys, including NF-kB, TNF-a, and IL-6. Additionally, vitamin K, beta-alanine, and glutathione metabolism are the main regulated metabolic pathways. Thus, lycopene-loaded bilosomes ameliorate high-fat diet-induced chronic nephritis in mice through the TLR4/MyD88 inflammatory pathway.

## Data Availability

Data is contained within the article.

## Supplementary Materials

The following supporting information can be downloaded at: https://www.mdpi.com/article/10.3390/foods11193042/s1.

## Abbreviations

Mice fed normal diet	Con group
Mice fed HFD diet	Mod group
Mice fed HFD diet and bilosomes	Bilo group
Mice fed HFD diet and lycopene-loaded bilosomes	Blio+Ly group
High-fat diet	HFD
Toll-like receptor 4	TLR4
myeloid differentiation primary gene 88	MyD88
nuclear factor kappa-B	NF-kB
interleukin-1β	IL-1β
interleukin-6	IL-6
tumor necrosis factor-a	TNF-a
tIR domain-coding adaptor protein	TIRAP
total internal reflection fluorescence	TIRF
principal component analysis	PCA
nuclear factor erythroid 2-related factor 2	Nrf2
fasting blood glucose	FBG
blood urea nitrogen	BUN
serum creatinine	SCr
microalbumin	MALB
a mixture of tris-buffered saline and Tween 20	TBST
sodium dodecyl sulfate polyacrylamide gel electrophoresis	SDS-PAGE
quantitative real-time PCR	qRT-PCR
gas chromatography–mass spectrometry	GC–MS
