# Supplementary material for: Lycopene-Loaded Bilosomes Ameliorate High-Fat Diet-Induced Chronic Nephritis in Mice through the TLR4/MyD88 Inflammatory Pathway
Source: Foods. 2022 Sep 30;11(19):3042. doi: 10.3390/foods11193042 (PMC9564075; doi:10.3390/foods11193042)
Supplement: Supplementary file 1 [file foods-11-03042-s001.zip › foods-1894933-supplementary.pdf]

foods

MDPI

**Supplementary Materials**

**Table S1.** Sequences of PCR primers.

| | Upstream primer (5′-3′) | Downstream primer (5′-3′) |
|---|---|---|
| β-actin | GTTGTCGACGACGAGGG | GCACAGAGCCTCGCCTT |
| TLR4 | CACCAGGAAGCTTGAATCCCT | TGCTGAGTTTCTGATCCATGC |
| NF-κB | TGCAACAGATGGGCTACACAGT | TCATCTATGTGCTGCCTCGT |
| MyD88 | CATACCCTTGGTCGCGCTTA | TCCGAGGGTTCAAGAACAGC |
| TRIF | GATCGTTTTCGCCGAGATGC | GTTGTACTTGTGCTGCCTGC |
| TIRAP | CGCTGGAGCAAAGACTACGA | CAGGAAGCAGCGTAGACTGG |
| IL-6 | GTTGCCTTCTTGGGACTGATGT | GGTCTGTTGTGGGTGGTATCCT |
| IL-1β | GGACAGAACATAAGCCAACA | CTTTCATCACACAGGACAGG |
| TNF-α | ATGGGCTCCCTCTCATCAGT | GCTTGGTGGTTTGCTACGAC |
